# Glycogen synthase kinase-3: a new therapeutic target in renal cell carcinoma

**DOI:** 10.1038/sj.bjc.6605437

**Published:** 2009-11-17

**Authors:** V Bilim, A Ougolkov, K Yuuki, S Naito, H Kawazoe, A Muto, M Oya, D Billadeau, T Motoyama, Y Tomita

**Affiliations:** 1 Department of Urology, Yamagata University School of Medicine, Iida-nishi 2-2-2, Yamagata 990-9585, Japan; 2 Department of Pathology, Feinberg School of Medicine, Northwestern University, Ward Building 3-140, 303 E. Chicago Avenue, Chicago, IL 60611, USA; 3 Department of Urology, Keio University School of Medicine, Shinano-machi 35, Shinjuku-ku, Tokyo, Japan; 4 Division of Oncology Research, Mayo Clinic College of Medicine, 200 First Street Southwest, Rochester, MN 55905, USA; 5 Department of Human Pathology (Second Department of Pathology), Yamagata University School of Medicine, Iida-nishi 2-2-2, Yamagata 990-9585, Japan

**Keywords:** renal cell carcinoma, apoptosis, glycogen synthase kinase-3*β*

## Abstract

**Background:**

Renal cell carcinoma (RCC) is highly resistant to chemotherapy because of a high apoptotic threshold. Recent evidences suggest that GSK-3*β* positively regulates human pancreatic cancer and leukaemia cell survival in part through regulation of nuclear factor (NF-*κ*B)-mediated expression of anti-apoptotic molecules. Our objectives were to determine the expression pattern of GSK-3*β* and to assess the anti-cancer effect of GSK-3*β* inhibition in RCC.

**Methods:**

Immunohistochemistry and nuclear/cytosolic fractionation were performed to determine the expression pattern of GSK-3*β* in human RCCs. We used small molecule inhibitor, RNA interference, western blotting, quantitative RT–PCR, BrDU incorporation and MTS assays to study the effect of GSK-3*β* inactivation on renal cancer cell proliferation and survival.

**Results:**

We detected aberrant nuclear accumulation of GSK-3*β* in RCC cell lines and in 68 out of 74 (91.89%) human RCCs. We found that pharmacological inhibition of GSK-3 led to a decrease in proliferation and survival of renal cancer cells. We observed that inhibition of GSK-3 results in decreased expression of NF-*κ*B target genes Bcl-2 and XIAP and a subsequent increase in renal cancer cell apoptosis. Moreover, we show that GSK-3 inhibitor and Docetaxel synergistically suppress proliferation and survival of renal cancer cells.

**Conclusions:**

Our results show nuclear accumulation of GSK-3*β* as a new marker of human RCC, identify that GSK-3 positively regulates RCC cell survival and proliferation and suggest inhibition of GSK-3 as a new promising approach in the treatment of human renal cancer.

Kidney cancer accounts for approximately 2–3% of all cancers worldwide. It is the seventh most common cancer and the tenth most common cause of cancer death in men and the ninth most common cause of cancer in women. In 2008, an estimated 54 000 adults in the United States have been diagnosed with renal cell carcinoma (RCC). Approximately 13 000 deaths from RCC have occurred in 2008 (Cancer.Net by ASCO). The 5-year survival rate for patients with metastatic RCC is less than 10% because of the tumours resistance to chemo- and radiotherapy. About one third of newly diagnosed RCC patients present with metastases and up to one half of patients develop metastatic disease during follow-up (Bukowski, 1997). Immunotherapy overall efficacy rate does not exceed 10–20% in RCC cases (Bukowski, 2001). Recently, molecular targeting drugs including multiple kinase inhibitors Sorafenib and Sunitinib (Motzer and Bukowski, 2006; Ljungberg *et al*, 2007) have been suggested as first-line treatment for metastatic RCC, although the treatment response is not long-standing and the RCC tumours inevitably progress. Thus, the identification of novel therapeutic targets in RCC is urgently needed.

There are diverse factors that contributes to RCC progression and chemoresistance, including activation of nuclear factor-*κ*B (NF-*κ*B; Oya *et al*, 2001, 2003; An *et al*, 2004). Increased expression of Bcl-2 and XIAP anti-apoptotic molecules, NF-*κ*B target genes, has an important function in renal cancer cell survival and chemoresistance (Bilim *et al*, 2008) and resistance to immunotherapy (Maruyama *et al*, 2006). Previous studies suggest a positive role for GSK-3*β* in the regulation of NF-*κ*B activity (Hoeflich *et al*, 2000; Ougolkov *et al*, 2005, 2007). GSK-3 is a pluripotent serine–threonine kinase with a numerous intracellular target proteins (Jope and Johnson, 2004). GSK-3 has two isoforms, *α* and *β*, which are coded by two different genes (Jope and Johnson, 2004). Previously, we showed that inhibition of GSK-3 resulted in apoptosis induction through decreased expression of NF-*κ*B target genes Bcl-2 and XIAP in chronic lymphocytic leukaemia (CLL) and pancreatic cancer cells (Ougolkov *et al*, 2005, 2007). It has been shown that efficient localisation of NF-*κ*B to the promoter of certain genes requires GSK-3*β* (Steinbrecher *et al*, 2005). Recently, we showed that GSK-3 contributes to the maintenance of active chromatin at NF-*κ*B target gene Bcl-2 and XIAP promoters, allowing p65 binding and transcriptional activation in cancer cells (Ougolkov *et al*, 2007).

Although our recent studies suggest GSK-3 as an important factor of NF-*κ*B-mediated cancer cell survival and proliferation in pancreatic cancer and CLL (Ougolkov *et al*, 2005, 2007), the role of GSK-3 in the proliferation, survival and chemoresistance of RCC is unknown. Here, for the first time, we show that genetic depletion or pharmacological inhibition of GSK-3 results in decreased renal cancer cell proliferation and survival. Moreover, we found abberant GSK-3*β* nuclear overexpression in RCC cell lines and most human renal carcinomas. Furthermore, we show a synergistic anti-cancer effect of GSK-3 inhibitor and Docetaxel in renal cancer cells. Our results suggest GSK-3 as a novel potential therapeutic target in the treatment of RCC.

## Materials and methods

### Patients and immunohistochemistry

The study was approved by the Ethical Committee of Yamagata University and all patients signed an informed consent form. Seventy-six surgical specimens from 75 unselected patients (1 patient with multiple tumours was operated twice) who underwent surgery (27 open, 49 laparoscopic; 56 radical nephrectomies, 20 nephron sparing surgeries, right 37, left 39) for RCC from 2003 to 2006 at the Yamagata University Hospital were included in the study. Patients' clinical characteristics are presented in the Table 1. The tumours were fixed in 10% buffered formalin and embedded in paraffin, and the samples were coded. Paraffin sections were routinely stained with haematoxylin and eosin and a pathological diagnosis was made. Pathological staging was determined according to the UICC TNM classification of malignant tumours. Pathological diagnosis for 2 tumours was oncocytoma and the remaining 74 were malignant tumours. Pathological grades were assigned according to a system developed by the Japanese Urological Association based on the degree of atypia of tumour cells.

Monoclonal mouse antibody for GSK-3*β* from BD Transduction (San Diego, CA, USA) or rabbit polyclonal antibody for anti-phospho-glycogen synthase (pGS) (#3891) from Cell Signaling Technology (Danvers, MA, USA) was used for immunohistochemical analysis. Immunohistochemical staining was performed as described earlier (Bilim *et al*, 2008). Two different sections from each tumour were examined by immunohistochemistry. For each staining, two 5 *μ*m-thick paraffin sections from different parts of each tumour representative of the entire tumour were mounted on silanised glass slides (Dako Japan, Tokyo, Japan). After deparaffination and rehydratation, epitops were reactivated by autoclaving sections in 10 mM citric buffer (pH 6.0) for 10 min. The slides were incubated with the primary antibodies overnight at 4°C in a moist chamber. After washing with PBS, bound antibody was detected by peroxidase method using Histofine simple stain MAX-PO MULTY (Nichirei, Tokyo, Japan). The staining reaction was developed by DAB in the presence of H_2_O_2_. Nuclear counterstaining was performed by haematoxylin. Positive and negative controls were included in each staining series. Positive immunohistochemical staining of GSK-3*β* or pGS in tumours confirmed by western immunoblotting served as a positive control. As a negative control, each primary antibody was replaced by either nonimmune mouse or rabbit immunoglobulin. The results were observed using Olympus (Tokyo, Japan) BX50 microscope equipped with Olympus DP12 digital microscope camera. All slides were evaluated for immunostaining without any knowledge of the clinical data. There were no inter- and intra-sample fluctuations in terms of the staining intensity. GSK-3*β* nuclear accumulation was defined as positive staining of >10% of cancer cell nuclei throughout the tumour regardless of cytoplasmic expression as we established earlier for this antibody (Ougolkov *et al*, 2006). Positive pGS expression was defined as positive staining of more than 80% of cancer cells throughout the tumour.

### Cell culture and reagents

Renal cell cancer cell lines ACHN, KRC/Y, Caki1, Caki2, A704, A498 and KH39 were purchased from ATCC (Manassas, VA, USA). KU19-20 was kindly provided by Dr Mototsugu Oya (Department of Urology, School of Medicine, Keio University, Tokyo, Japan). The cells were cultured as described earlier (Bilim *et al*, 2000). GSK-3 inhibitor AR-A014418 was purchased from Calbiochem (San Diego, CA, USA). AR-A014418 (thiazole-containing urea compound), a small molecule inhibitor, inhibits GSK-3 in an ATP-competitive manner (*in vitro* IC_50_=104 nM) and does not significantly inhibit cdk or other 26 kinases showing high specificity for GSK-3 (Bhat *et al*, 2003). Other two GSK-3 inhibitors, SB-216763 (ATP-competitive, arylindolemaleimide) and TDZD8 (non-ATP-competitive, thiadiazolidinone derivative), were purchased from Cayman Chemicals (Ann Arbor, MI, USA) and Sigma-Aldrich Japan (Tokyo, Japan), respectively. SB-216763 inhibits GSK-3 *in vitro* with an IC_50_ value of less than 100 nM with no significant inhibition of 24 other protein kinases (Coghlan *et al*, 2000). TDZD8, a potent inhibitor of GSK-3 (IC_50_=2 *μ*M), did not inhibit protein kinases A or C, CK-2 or CDK1/cyclin B kinases at >100 *μ*M (Martinez *et al*, 2002). Docetaxel was from Sigma-Aldrich Japan.

### Immunoblotting

Immunoblotting was performed as described earlier (Bilim *et al*, 2000). HRP-labelled second antibody was detected using a SuperSignal West Pico Substrate (Pierce, Rockford, IL, USA) according to the manufacturer's instructions. *β*-Actin was used as a loading control. The images were analysed using *UN-SCAN-Itgel* Automated Digitizing System software (version5.1 for Windows, Silk Scientific Inc., Orem, UT, USA). The following antibodies were used: anti-Bcl-2 (clone 124, DAKO, Japan), anti-glycogen synthase (GS) (#3893), anti-pGS (#3891) from Cell Signaling Technology; anti-GSK-3*β* (clone 7), anti-PARP (clone 7D3-6), anti-NF-*κ*B (p65) (clone 20), anti-XIAP (clone 28) from BD Transduction; anti-GSK-3*α* (#07-389) from Upstate Cell Signaling Solutions (Lake Placid, NY, USA); and anti-*β*-actin from Abcam Inc. (Cambridge, MA, USA). Nuclear/cytosolic fractionation was performed by modified Dignam method as described earlier (Ougolkov *et al*, 2006).

### RNA extraction and real-time RT–PCR

Total cellular RNA was extracted using the SV total RNA Isolation System (Promega, Madison, WI, USA) and the first-strand DNA was synthesised using a cDNA Reverse Transcription kit (Applied Biosystems Japan, Tokyo, Japan) following the manufacturer's instructions. Real-time quantitative RT–PCR was performed in the 7300 Real-Time PCR System (Applied Biosystems). We used pre-designed TaqMan Gene Expression Assays (Applied Biosystems) targeting human *Bcl-2* (Hs00236808_s1), *XIAP* (Hs00236913_m1) mRNA and *GAPDH* (4352934E) mRNA as an endogenous control. Each experiment was repeated at least three times to confirm reproducibility with the reaction in triplicate wells for each sample using a TaqMan Universal PCR Master Mix (Applied Biosystems) according to the standard protocol. The expression of the target mRNA was quantified relative to that of the *GAPDH* mRNA and untreated controls were used as a reference.

### Chromatin immunoprecipitation assay

Chromatin immunoprecipitation (ChIP) was performed as described earlier (Ougolkov *et al*, 2007). Briefly, ACHN cells were treated with 50 *μ*M of AR-A014418 or control DMSO for 48 h. After that the cells were cross-linked with formaldehyde for 15 min at room temperature and immunoprecipitated using the Chromatin Immunoprecipitation kit (Upstate Biotechnology, Lake Placid, NY, USA) according to the manufacturer's instructions. Anti-NF-*κ*B p65 antibody was from Santa Cruz Biotechnology (Santa Cruz, CA, USA). One hundred and six bps of the XIAP promoter and 168 bps of the Bcl-2 promoter were detected in immunoprecipitated samples by PCR. PCR products were separated on a 2% agarose gel and visualised under UV light after staining with ethidium bromide.

### RNA interference

Genetic knockdown of GSK-3*β* and GSK-3*α* was achieved using Validated Stealth RNAi DuoPak (Invitrogen Japan, Tokyo, Japan). Unrelated control siRNA (Invitrogen) was also used. Transfection was carried out using Lipofectamine 2000 (Invitrogen) according to manufacturer's recommendations.

### Measurement of cell viability, proliferation and apoptosis

Cell viability was detected with a colorimetric assay, the CellTiter 96 AQueous One Solution Cell Proliferation Assay (Promega, Madison, WI, USA) using tetrazolium compound according to the manufacturer's instructions as described earlier (Bilim *et al*, 2008). For estimation of cell proliferation BrdU cell proliferation assay (Calbiochem) was applied according to the manufacturer's instructions as described earlier (Bilim *et al*, 2008). For detection of apoptotic morphology, cells were cultured in Lab-Tek Chambers (Nunc Inc., Naperville, IL, USA), treated with AR-A014418. Apoptotic morphological changes were detected with Hoechst 33342 (Dojindo Laboratories, Kumamoto, Japan) staining followed by observation under fluorescence microscope Axiovert 200 (Carl Zeiss Japan, Tokyo, Japan). PI staining of the fixed cells, as described elsewhere, was applied for quantification of the late apoptotic events (sub-G1 fraction). Stained cells were analysed on FACSCalibur Flow Cytometer (BD).

### Statistical analysis

Continuous variables are presented as the mean±s.d. All continuous variables in this study met the criteria for a normal distribution and were assumed to be parametric. They were analysed using a two tailed *t*-test or one-way ANOVA where appropriate with the post test for a linear trend. Associations between immunohistochemical staining and pathological or clinical characteristics were analysed using Fisher's exact test. Two-sided tests were used. Data were analysed using GraphPad Prism software package for Windows (GraphPad Software Inc., San Diego, CA, USA).

## Results

### GSK-3*β* is expressed and active in human renal cancer cells

**Figure 1:**
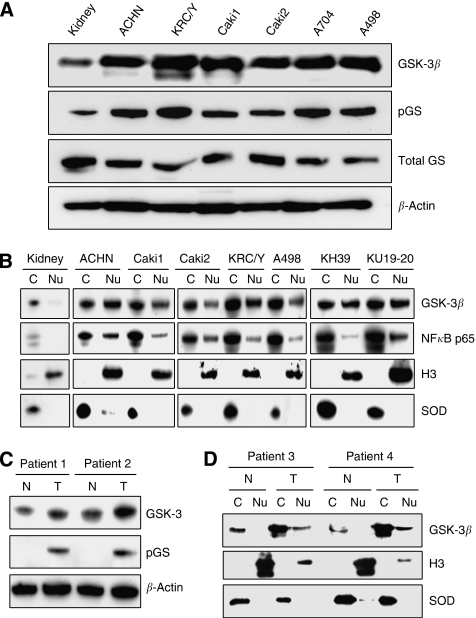
GSK-3*β* is overexpressed in nuclei of renal cancer cells. (**A**) Protein lysates from the indicated RCC cell lines and normal kidney as a control were separated by SDS–PAGE (50 *μ*g per well), transferred to PVDF membrane and probed with antibodies against GSK-3*β*, phospho-glycogen synthase (pGS) and total glycogen synthase (GS). (**B**) Cytosolic (C) and nuclear (Nu) fractions were prepared from RCC cell lines and normal kidney, separated by SDS–PAGE (50 *μ*g per well), transferred to PVDF membrane and probed with indicated antibodies. Cu/Zn supeoxide dismutase (SOD) and histone H3 (H3) were used as cytosolic and nuclear markers, respectively. (**C**) Expression of GSK-3*β* and pGS was detected in protein extracts from primary tumour (T) and corresponding normal kidney tissue (N) obtained from kidney cancer patients. (**D**) Nuclear (Nu) and cytosolic (C) fractions were prepared from fresh tumour (T) and corresponding normal kidney tissue (N) sampled from kidney cancer patients, and analysed as described in (**B**).

Using western blotting, we detected higher levels of GSK-3*β* expression in RCC cell lines compared with normal kidney (Figure 1A). We also found higher levels of phosphorylation of GS (pGS), a primary GSK-3 substrate, in RCC cell lines compared with normal kidney suggesting that GSK-3 is active in renal cancer cells (Figure 1A). Using paired samples of tumour and normal kidney tissues from renal cancer patients, we found phosphorylation of GS only in tumour tissues but not in its normal counterparts suggesting higher activity of GSK-3 in human RCCs (Figure 1C). Moreover, we found that expression of GSK-3*β* was higher in tumour compared with corresponding normal kidney tissue (Figure 1C). These data indicate that high levels of GSK-3*β* expression and activity are features of RCC.

### GSK-3*β* is accumulated in the nucleus of renal cancer cells

GSK-3*β* has been shown as positive regulator of NF-*κ*B-mediated survival and proliferation of cancer cells (Ougolkov *et al*, 2005, 2007; Wilson and Baldwin, 2008). Recently, we have shown aberrant nuclear accumulation of GSK-3*β* in pancreatic cancer and leukaemia cells (Ougolkov *et al*, 2006, 2007). It has been suggested that nuclear GSK-3*β* might contribute to NF-*κ*B-mediated expression of anti-apoptotic molecules and cancer cell survival (Ougolkov *et al*, 2006, 2007). We found that high levels of GSK-3*β* expression and activity are features of RCC (Figure 1A and C). However, the subcellular localisation of GSK-3*β* in renal cancer cells is unknown.

Using nuclear/cytoplasmic fractionation, we found aberrant nuclear expression of GSK-3*β* in human renal carcinomas but not in their normal counterparts (Figure 1D). Moreover, the levels of cytoplasmic GSK-3*β* in human renal carcinomas were higher than in normal kidney tissues (Figure 1D). Nuclear accumulation of GSK-3*β* and NF-*κ*B p65 was detected in seven RCC lines: KH39, KU19-20, ACHN, Caki1, Caki2, KRC/Y and A498 (Figure 1B) and was undetectable in normal kidney (Figure 1B).

**Figure 2:**
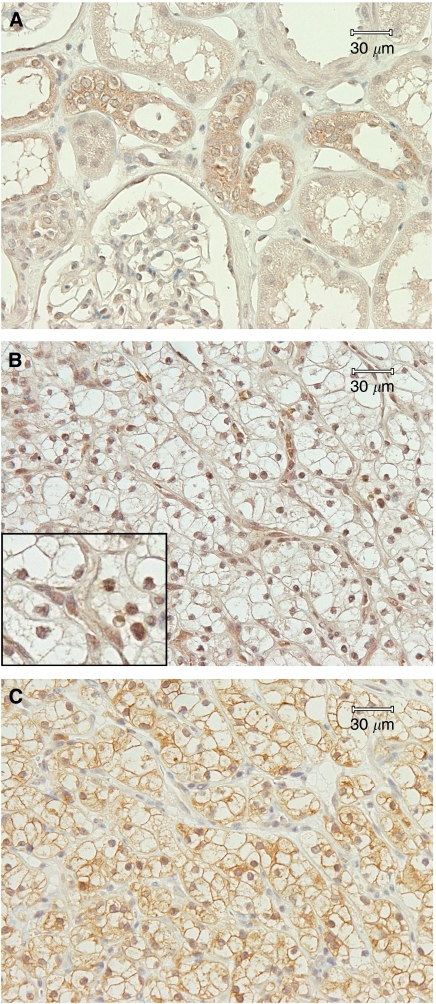
Immunohistochemical analysis of GSK-3*β* expression in normal human kidney (**A**). Immunohistochemical analysis of GSK-3*β* (**B**) and pGS (**C**) expression in serial sections of renal carcinoma. Insert in (**B**) shows higher magnification view.

Using immunohistochemical staining, we found weak cytoplasmic expression of GSK-3*β* in a fraction of glomerular and tubular epithelial cells in normal kidney (Figure 2A). It is interesting to note that oncocytomas, which are benign kidney tumours, showed only cytoplasmic expression of GSK-3*β* and no pGS was detected in these tumours. We found aberrant nuclear accumulation of GSK-3*β* in 68 out of 74 (92%) human RCCs (Figure 2B; Table 2). Sixty-nine (90.79%) tumours were positive for pGS (Figure 2C; Table 2). Nuclear accumulation of GSK-3*β* correlated with pGS positivity (Fisher's exact test *P*=0.0017, *χ*^2^ with Yate's correction *P*=0.0004), which indicates GSK-3*β* active state. Clear cell RCC subtype is associated with worse survival in RCCs (Beck *et al*, 2004). We found that clear cell RCC was significantly associated with aberrant GSK-3*β* nuclear accumulation (Fisher's exact test *P*=0.0185, *χ*^2^ with Yate's correction *P*=0.0219) and pGS positivity (Fisher's exact test *P*=0.0008, *χ*^2^ with Yate's correction *P*=0.0002). GSK-3*β* nuclear accumulation correlated with neither stage nor grade in RCCs and it was observed equally frequently in low and high stages and grades (Table 2). Our results suggest that aberrant nuclear accumulation of GSK-3*β* is a feature of renal cancer cells and GSK-3*β* activation might be a critical early step of RCC carcinogenesis.

### Pharmacological inhibition and genetic depletion of GSK-3 decrease proliferation and survival of renal cancer cells

**Figure 3:**
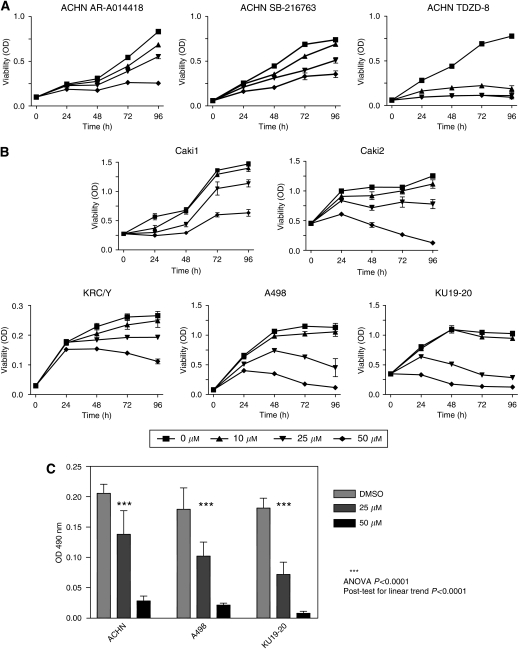

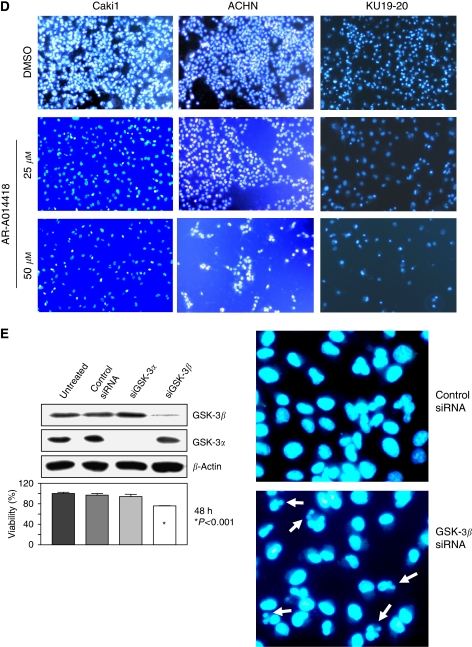
Inhibition of GSK-3 suppresses proliferation of renal cancer cells. (**A**) Relative cell viability was measured by MTS assay in ACHN renal cancer cell line treated with indicated doses of AR-A014418, SB-216763 or TDZD-8 for 24, 48, 72 and 96 h. (**B**) Relative cell viability was measured by MTS assay in RCC cell lines treated with indicated doses of AR-A014418 for 24, 48, 72 and 96 h. (**C**) ACHN, A498 and KU19-20 renal cancer cells were treated with diluent (DMSO) or AR-A014418 with indicated doses for 48 h. BrdU colometric assay was performed as described in ‘Materials and Methods’. The results are presented as OD 490 nm (ANOVA *P*<0.0001, post test for linear trend *P*<0.0001). (**D**) ACHN, Caki1 and KU19-20 renal cancer cells were cultured in the presence of DMSO or indicated concentrations of AR-A014418 for 96 h, followed by Hoechst 33342 staining. (**E**) ACHN renal cancer cells were transfected with control siRNA, GSK-3*β* or GSK-3*α* siRNA using Lipofectamine; 48 h after transfection, relative cell viability was measured in transfected cancer cells by MTS assay as shown in lower panel. Western blot for GSK-3*α*, GSK-3*β* and actin as control for loading is presented in the upper panel. Right panel represents Hoechst 33342 staining of ACHN cells transfected with control siRNA (right-upper) or GSK-3*β* siRNA (right-lower). Apoptotic cells are indicated by arrows.

Although our recent studies suggest GSK-3 as an important factor of NF-*κ*B-mediated cancer cell survival and proliferation in pancreatic cancer and CLL (Ougolkov *et al*, 2006, 2007), the role of GSK-3 in the proliferation and survival of RCC is unknown. To determine whether active GSK-3 is essential for RCC cell survival and proliferation, first we tested the effect of three chemically distinct small molecule inhibitors of GSK-3: AR-A014418 (ATP-competitive) (Bhat *et al*, 2003), SB-216763 (ATP-competitive) (Coghlan *et al*, 2000), and TDZD8 (non-ATP-competitive) (Martinez *et al*, 2002) in ACHN renal cancer cells (Figure 3A). We found that all three distinct GSK-3 inhibitors can decrease viability of ACHN renal cancer cells (Figure 3A). Subsequently, we tested the anti-cancer effect of GSK-3 inhibitor AR-A014418 using six renal cancer cell lines, KH39, KU19-20, Caki1, Caki2, KRC/Y and A498. AR-A014418 is a potent and specific GSK-3 inhibitor described earlier (Bhat *et al*, 2003). We found that inhibition of GSK-3 decreased renal cancer cell viability in a dose- and time-dependent manner (Figure 3B). Using BrDU incorporation assay, we found that pharmacological inhibition of GSK-3 suppresses proliferation of renal cancer cells (Figure 3C). Using Hoechst staining, we found a dose-dependent induction of apoptosis in AR-A014418-treated renal cancer cells (Figure 3D). These results suggest that GSK-3 is a positive regulator of renal cancer cell proliferation and survival.

To determine whether the inhibitory effect on renal cancer cell survival by pharmacological inhibition of GSK-3 was specific to GSK-3*β*, we depleted GSK-3*α* or GSK-3*β* expression in ACHN cancer cells using siRNA (Figure 3E). We found that depletion of GSK-3*β* leads to a significant decrease in renal cancer cell survival accompanied with apoptotic morphological changes as detected by Hoechst staining, whereas depletion of GSK-3*α* does not affect cancer cells (Figure 3E). These results suggest that GSK-3*β* is a selective regulator of survival in renal cancer cells.

**Figure 4:**
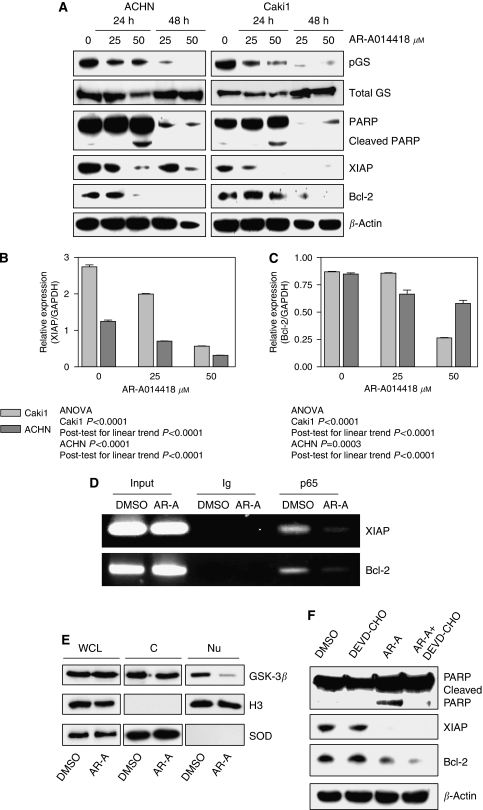
Inhibition of GSK-3 decreases expression of anti-apoptotic XIAP and Bcl-2 and induces apoptosis in renal cancer cells. (**A**) ACHN and Caki1 renal cancer cells were treated with 25 or 50 *μ*M of AR-A014418; 24 and 48 h after treatment, the cell pellet was collected, cell lysates were separated by SDS–PAGE (50 *μ*g per well), transferred to PVDF membrane and probed with indicated antibodies. pGS, phospho-glycogen synthase. (**B**, **C**) ACHN and Caki1 renal cancer cells were treated with 25 or 50 *μ*M of AR-A014418; 24 h after treatment, the cell pellet was collected and RNA was extracted. Relative expression (target gene value normalised by GAPDH) of XIAP (**B**) and Bcl-2 (**C**) genes was measured by real-time PCR using TaqMan probe technique as described in ‘Materials and Methods’. *P*-values of ANOVA and post test for linear trend are indicated. (**D**) Using chromatin immunoprecipitation (ChIP) assay, binding of NF-*κ*B p65 to the promoters of its target genes XIAP and Bcl-2 was evaluated with in ACHN RCC cells treated with DMSO or 50 *μ*M AR-A014418 (AR-A) for 48 h. (**E**) ACHN renal cancer cells were treated with either DMSO or AR-A014418 (50 *μ*mol l^−1^) for 24 h, nuclear/cytosolic fractions were prepared and cytozolic/nuclear GSK-3*β* protein was analysed as described in Figure 1B and D. (**F**) A498 renal cancer cells were treated with diluent (DMSO), DEVD-CHO (caspase inhibitor), AR-A014418 (AR-A) or AR-A014418+DEVD-CHO; 24 h after treatment, the cell pellet was collected and protein expression analysis was performed as described in (**A**).

Using western blotting, we estimated the level of GSK-3 inhibition by detection of the level of pGS, a primary GSK-3 substrate (Figure 4A). We found that treatment of ACHN and Caki1 renal cancer cells with different concentrations of AR-A014418 resulted in a dose- and time-dependent inhibition of GSK-3 activity, as measured by the levels of pGS (Figure 4A). We found that inhibition of GSK-3 induces dose- and time-dependent apoptosis (as measured by PARP cleavage) in ACHN and Caki1 renal cancer cells (Figure 4A). Consistently, using Hoechst staining (Figure 3D) and flow cytometry (data not shown), we found a dose-dependent increase in the number of apoptotic cells in AR-A014418-treated ACHN and Caki1 renal cancer cells. These results suggest that inhibition of GSK-3 decreases survival of renal cancer cells.

Multiple factors contribute to RCC progression, including activation of NF-*κ*B (Oya *et al*, 2001, 2003; An *et al*, 2004). Increased expression of Bcl-2 and XIAP anti-apoptotic molecules, NF-*κ*B target genes has an important function in renal cancer cell survival (Tomita *et al*, 1996; Maruyama *et al*, 2006; Bilim *et al*, 2008). As GSK-3*β* has a positive role in expression of certain NF-*κ*B-regulated genes (Hoeflich *et al*, 2000; Ougolkov *et al*, 2005, 2007), we investigated whether inhibition of GSK-3 affects NF-*κ*B-mediated expression of Bcl-2 and XIAP in renal cancer cells. Using western immunoblotting, we found that inhibition of GSK-3 resulted in a significant decrease in the expression of anti-apoptotic proteins Bcl-2 and XIAP in ACHN and Caki1 renal cancer cells (Figure 4A). Using real-time PCR, we found that inhibition of GSK-3 resulted in a marked reduction in the expression of NF-*κ*B target genes Bcl-2 and XIAP, suggesting a downregulation of NF-*κ*B transcriptional activity in renal cancer cells (Figure 4B and C).

To unveil the potential mechanism of XIAP and Bcl-2 transcriptional suppression by GSK-3 inhibition, we immunoprecipitated chromatin with anti-p65 antibody in a ChIP assay. Accessibility of XIAP and Bcl-2 promoters by NF-*κ*B p65 was drastically decreased on GSK-3 inhibition (Figure 4D). Consistent with previous findings in pancreatic cancer and leukaemia cells (Ougolkov *et al*, 2006, 2007), we found that pharmacologic inhibition of GSK-3*β* resulted in depletion of nuclear GSK-3*β* from the renal cancer cells' nuclei by 24 h of AR-A014418 treatment (Figure 4E). However, nuclear NF-*κ*B p65 levels were not changed (data not shown). The data are in agreement with the hypothesis that GSK-3*β* positively modifies NF-*κ*B transcriptional activity downstream to the IKK complex.

To determine whether Bcl-2 and XIAP downregulation was a cause or a consequence of caspase activation and apoptosis, we treated A498 renal cancer cells with DMSO, AR-A014418, DEVD-CHO (reversible tetrapeptide inhibitor of caspase-3 and caspase-7) or a combination of AR-A014418 and DEVD-CHO (Figure 4F). We found that DEVD-CHO could rescue the apoptotic effect of GSK-3 inhibition by AR-A014418, whereas DEVD-CHO did not affect the decrease in Bcl-2 and XIAP protein levels in AR-A014418-treated cells (Figure 4F). These results suggest that downregulation of Bcl-2 and XIAP expression in AR-A014418-treated renal cancer cells occurs upstream of caspase activation.

Taken together, our results suggest that inhibition of GSK-3 suppresses the expression of NF-*κ*B target genes Bcl-2 and XIAP, resulting in decreased survival of renal cancer cells.

### AR-A014418 and Docetaxel synergistically suppress survival of renal cancer cells

**Figure 5:**
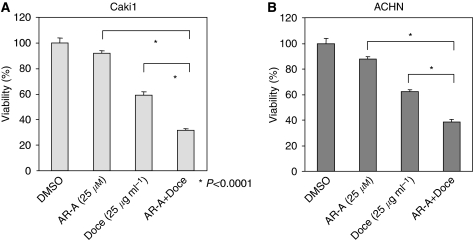
GSK-3 inhibitor and Docetaxel synergistically suppress viability of renal cancer cells. Relative cell viability was measured by MTS assay in Caki1 (**A**) and ACHN (**B**) renal cancer cells treated with 25 *μ*M of AR-A014418, 25 *μ*g ml^−1^ Docetaxel or a combination of both for 24 h. Combined treatment with AR-014418 and Docetaxel significantly suppressed cancer cell viability (*P*<0.0001) compared with both agents, and the effect of combination of the two drugs was synergistic.

The 5-year survival rate for patients with metastatic RCC is less than 10% (Motzer *et al*, 1996) because of the tumours resistance to chemo- and radiotherapy. Chemotherapeutic effect for RCC is very limited because kidney cancer is intrinsically chemoresistant. There are diverse factors that contribute to RCC chemoresistance, including activation of NF-*κ*B (Oya *et al*, 2001, 2003; An *et al*, 2004). Increased expression of Bcl-2 and XIAP anti-apoptotic molecules, NF-*κ*B target genes, has an important function in renal cancer cell survival and chemoresistance (Bilim *et al*, 2008). In this study, we show that inhibition of GSK-3 suppresses NF-*κ*B-mediated expression of Bcl-2 and XIAP leading to a decreased survival of renal cancer cells. To determine whether inhibition of GSK-3 could be useful in combination with conventional chemotherapeutic agent in the treatment of RCC, we treated renal cancer cells with AR-A014418 and Docetaxel, a well-established chemotherapeutic drug. Docetaxel has a limited cytotoxic effect in clinical RCC (Hartmann and Bokemeyer, 1999). We found that inhibition of GSK-3 sensitised ACHN and Caki1 cancer cells to Docetaxel, leading to a significant decrease in survival of renal cancer cells (Figure 5). Our results suggest that the combination of GSK-3 inhibitor with Docetaxel could be a superior treatment for human RCC.

## Discussion

Recent studies show that GSK-3*β* has an important function in pathogenesis of human cancer, including leukaemia (Ougolkov *et al*, 2007; Wang *et al*, 2008), pancreatic (Ougolkov *et al*, 2005, 2006), prostate (Mazor *et al*, 2004; Sun *et al*, 2007), colorectal (Shakoori *et al*, 2005), ovarian (Cao *et al*, 2006), thyroid (Kunnimalaiyaan *et al*, 2007) and brain (Kotliarova *et al*, 2008) carcinomas. However, the role of GSK-3*β* in kidney cancer remains unknown.

In this study, we identify GSK-3 as a positive regulator of RCC cell survival, proliferation and chemoresistance. We found GSK-3*β* aberrant nuclear accumulation in most (91.89%) of human renal carcinomas, whereas GSK-3*β* was detectable only in cytoplasm in normal kidney tissue. Our results suggest nuclear accumulation of GSK-3*β* as a potential oncomarker of RCC. Our findings are supported by previous studies showing nuclear overexpression of GSK-3*β* in pancreatic cancer (Ougolkov *et al*, 2006) and CLL (Ougolkov *et al*, 2007). Immunohistochemical detection of GSK-3*β* nuclear accumulation could be a useful diagnostic method for pathological verification of kidney cancer.

It has been suggested that GSK-3*β* is directed to the nucleus by releasing of its nuclear localisation signal from cytosolic complexes (Meares and Jope, 2007). Recently, we have shown that only active form of GSK-3*β* is detectable in the nucleus of pancreatic cancer cells (Ougolkov *et al*, 2006). Although inactive form of GSK-3*β* is able to translocate to the nucleus from cytoplasm, it is rapidly degraded by proteasomal pathway within the nucleus of the cancer cell (Ougolkov *et al*, 2006). Whether GSK-3*β* kinase activity is required for its stabilisation in the nucleus of renal cancer cells remains to be investigated.

Here, we show that inhibition of GSK-3 suppresses proliferation and survival of renal cancer cells. Our data are in agreement with other studies showing that inhibition of GSK-3 results in decreased proliferation and/or survival of CLL (Ougolkov *et al*, 2007), pancreatic (Ougolkov *et al*, 2005), colorectal (Shakoori *et al*, 2005), ovarian (Cao *et al*, 2006), thyroid (Kunnimalaiyaan *et al*, 2007) and brain (Kotliarova *et al*, 2008) cancer cells. We also observed retardation of tumour growth by GSK-3 pharmacological inhibition in mice xenograft model using RCC cell lines (manuscript in preparation). Our work suggests that inhibition of GSK-3 is a promising new approach to renal cancer therapy.

Multiple factors contribute to RCC progression, including activation of NF-*κ*B (Oya *et al*, 2001, 2003; An *et al*, 2004). Previous studies suggest a positive role for GSK-3*β* in the regulation of NF-*κ*B-mediated cancer cell survival (Ougolkov *et al*, 2005, 2007). Previously, we showed that inhibition of GSK-3 resulted in apoptosis induction through decreased expression of NF-*κ*B target genes Bcl-2 and XIAP in CLL and pancreatic cancer cells (Ougolkov *et al*, 2005, 2006, 2007). Increased expression of Bcl-2 and XIAP anti-apoptotic molecules, NF-*κ*B target genes, has an important function in renal cancer cell survival (Maruyama *et al*, 2006; Bilim *et al*, 2008). In this study, we show that inhibition of GSK-3 suppresses NF-*κ*B-mediated expression of Bcl-2 and XIAP leading to a decreased survival of renal cancer cells. Moreover, we show that depletion of GSK-3*β* by siRNA leads to a decrease in renal cancer cell survival, suggesting that GSK-3*β*, but not GSK-3*α*, is a selective regulator of survival in renal cancer cells.

Our finding of nuclear accumulation of GSK-3*β* suggests the possibility that GSK-3*β* could positively regulate NF-*κ*B-mediated transcriptional activation of Bcl-2 and XIAP in the nucleus of renal cancer cells. We show that pharmacological inhibition of GSK-3 resulted in depletion of its nuclear pool and decreased transcription of Bcl-2 and XIAP. Consistent with our recent study suggesting that GSK-3 may regulate the nuclear activity of NF-*κ*B in leukaemia cells by affecting the binding of p65/p50 to the promoters of NF-*κ*B target genes Bcl-2 and XIAP (Ougolkov *et al*, 2007), we found that GSK-3 positively regulates NF-*κ*B p65 binding to Bcl-2 and XIAP promoters in human renal cancer cells.

In renal carcinoma, NF-*κ*B activity could be boosted by chemotherapeutic stress, leading to tumour chemoresistance. Increased expression of Bcl-2 and XIAP anti-apoptotic molecules, NF-*κ*B target genes, has an important function in renal cancer cell survival and chemoresistance. Inactivation of NF-*κ*B can make renal cancer cells more sensitive to chemotherapy. As GSK-3*β* is a positive regulator of NF-*κ*B activity (Ougolkov *et al*, 2005, 2007), inhibition of GSK-3 may sensitise renal cancer cells to conventional chemotherapeutic agents. Here, we found that inhibition of GSK-3 suppresses NF-*κ*B-mediated expression of Bcl-2 and XIAP leading to a decreased survival of renal cancer cells. Moreover, we show that inhibition of GSK-3 sensitised kidney cancer cells to Docetaxel suggesting that GSK-3 might contribute to renal cancer chemoresistance. Our findings are supported by another study showing that GSK-3*β* positively regulates NF-*κ*B-mediated chemoresistance in acute myeloid leukaemia (De Toni *et al*, 2006).

Recently, it has been shown that GSK-3*β* inhibition enhanced Sorafenib-induced apoptosis in melanoma cells (Panka *et al*, 2008). As this combination potentially could be applied for the treatment of RCC we performed series of experiments. We also observed synergistic effect of AR-A014418 and Sorafenib to induce apoptosis in RCC *in vitro* and explored the underlying molecular mechanisms (manuscript in preparation).

Taken together, our work identifies GSK-3*β* as a novel potential therapeutic target in RCC and suggests the combination of GSK-3 inhibitors and standard chemotherapy could be a superior treatment for human RCC.

## Figures and Tables

**Table 1:** Patients' characteristics

Median age (range) years	59.5 (28–83)
Male/female	50/25
	
*Histological type*	
Oncocytoma	2
Malignant tumours	
Clear cell	64
Papillary	4
Chromophobe	3
Unclassified RCC	3
	
*pT stage*	
1a	34
1b	19
2	7
3a (including one adrenal involvement)	10
3b	4
	
*Grade*	
1	27
2	43
3	4

Abbreviation: RCC=renal cell carcinoma.

**Table 2:** Results of immunohistochemical study for GSK-3*β* and pGS

	**Total**	**GSK-3*β* nuclear**	**pGS positive**
*Histological type*			
Oncocytoma	2	0	0
Clear cell	64	60^a^	62^b^
Other	10	8	7
			
*pT stage (malignant tumours only)*			
1	53	50	52
2	7	6	7
3	14	12	10
			
*Grade (malignant tumours only)*			
1	27	26	27
2	43	39	39
3	4	3	3
Total	74 RCCs and 2 oncocytomas	68	69

Abbreviations: pGS=phospho-glycogen synthase; RCC=renal cell carcinoma.

a Fisher's exact test *P*=0.0185, *χ*^2^ with Yate's correction *P*=0.0219.

b Fisher's exact test *P*=0.0008, *χ*^2^ with Yate's correction *P*=0.0002.
